# Supplementary figures and images for: Persistent effects of acute trauma on Pavlovian-to-instrumental transfer
Source: Front Behav Neurosci. 2022 Oct 31;16:1028262. doi: 10.3389/fnbeh.2022.1028262 (PMC9659590; doi:10.3389/fnbeh.2022.1028262)

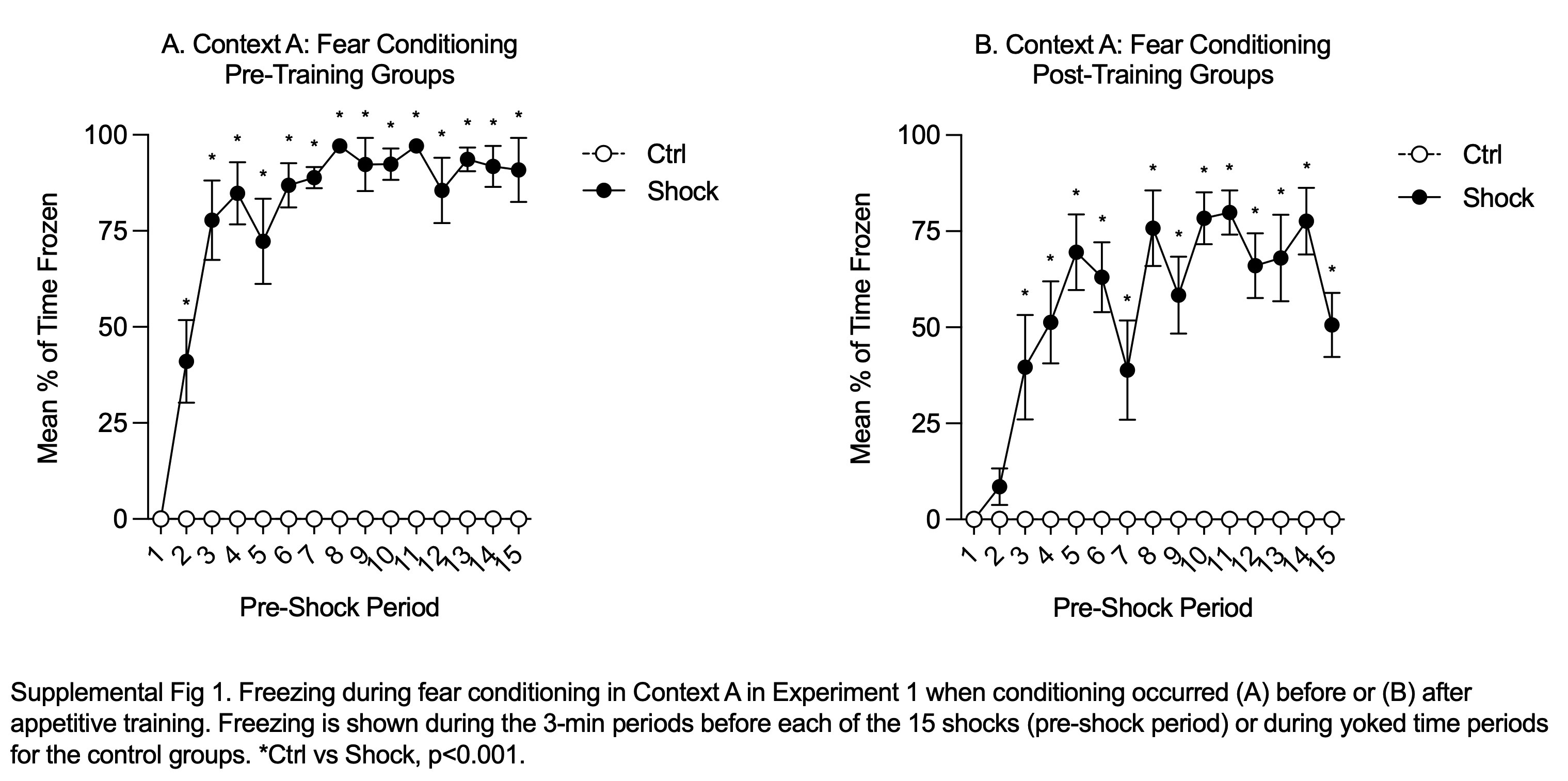

Supplement: Supplementary file 1 [file Image_1.JPEG]

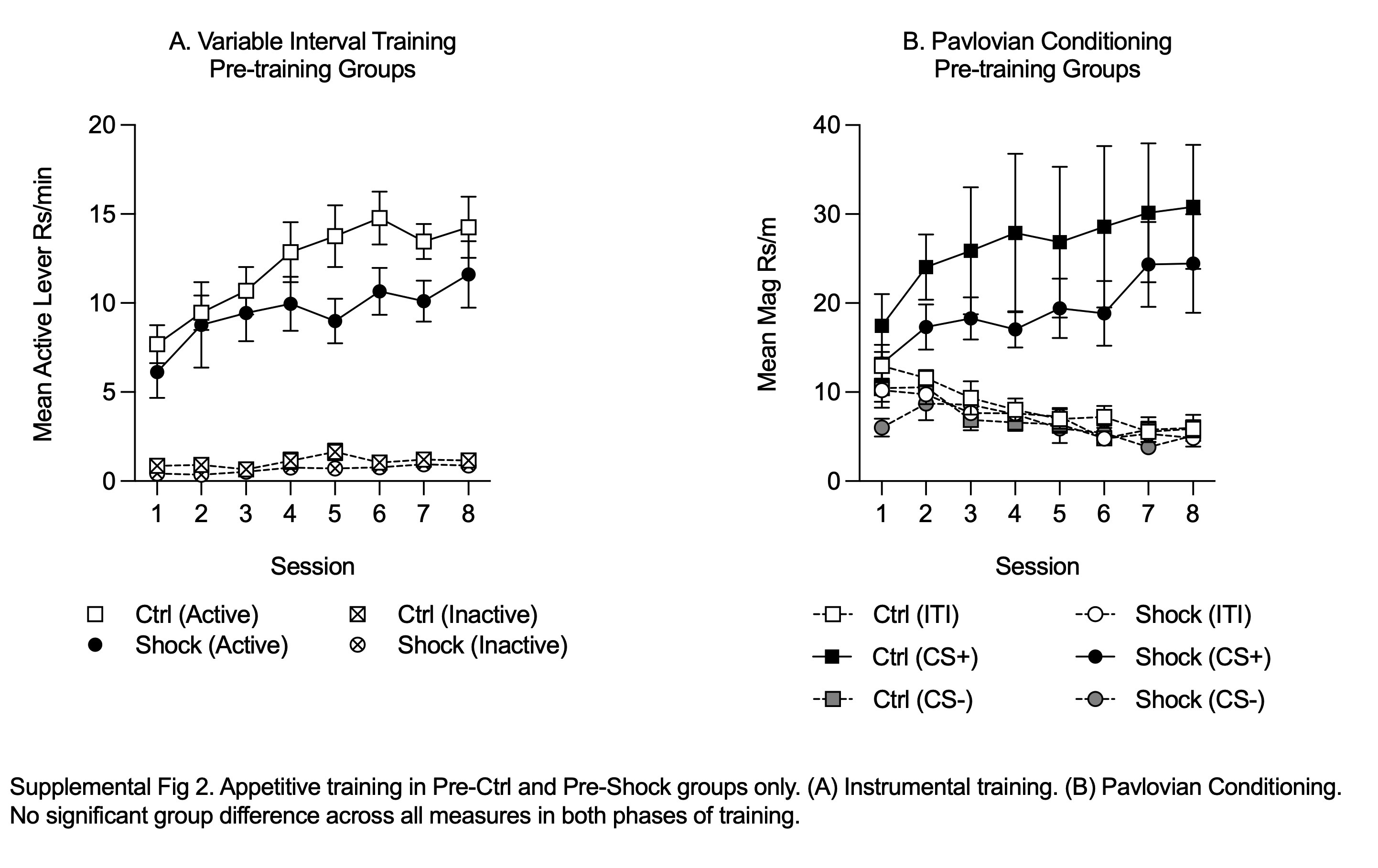

Supplement: Supplementary file 2 [file Image_2.JPEG]

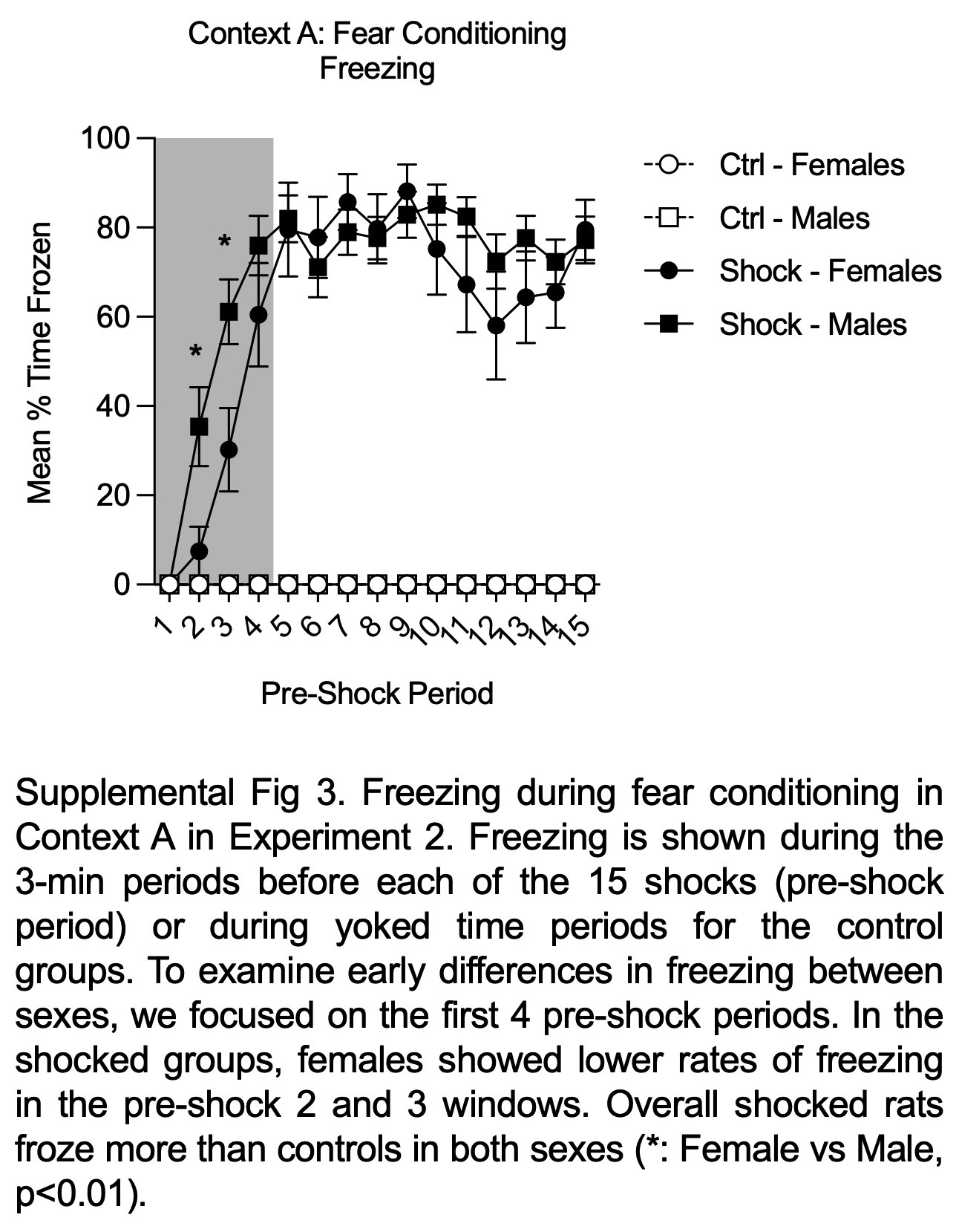

Supplement: Supplementary file 3 [file Image_3.JPEG]
